# Audit of Melatonin Use Across Child and Adolescent Mental Health Services (CAMHS) in Lincolnshire

**DOI:** 10.1192/bjo.2025.10535

**Published:** 2025-06-20

**Authors:** Ifetoluwanimi Adedapo, Emeka Onochie, Suneetha Siddabattuni

**Affiliations:** 1 Lincolnshire Partnership NHS Foundation Trust, Lincoln, United Kingdom; 2 Nottinghamshire Healthcare NHS Foundation Trust, Nottingham, United Kingdom; 3 Lincolnshire Partnership NHS Foundation Trust, Grantham, United Kingdom

## Abstract

**Aims:** To establish baseline data on melatonin use.

To compare the patterns of use with national guidelines.

To make recommendations to the teams.

**Methods:** A retrospective audit of patient records under the CAMHS services in Lincolnshire was undertaken to identify patients on melatonin as of June 2024. Data was collected from medical records between June and July 2024. Patients under 19 years and prescribed melatonin were included. Patients previously on melatonin but discontinued by June 2024 were excluded.

This audit was inspired by the POMH melatonin audit.

**Results:** 54 patients were identified, 23 males and 31 females. About half of the patients had been on melatonin for over one year (n=25).

Autism/autistic spectrum disorder was the most common diagnosis/comorbidity – 36 patients, 29 patients had an anxiety disorder, 21 patients had diagnosed/comorbid hyperkinetic disorders, 12 patients had mood disorders while 14 patients did not have a diagnosed neurodevelopmental disorder.

In 84.6% of prescriptions, evidence-based non-pharmacological measures were tried first.

The target symptom(s) for melatonin treatment was clear in 55.6% of cases. Sleep latency was the most common target, followed by reducing night-time awakening.

Licensed melatonin preparation was used in 46.3% of prescriptions. The preparation was however not clearly documented in most of the cases. (Licensed use covers insomnia with autism spectrum disorder (Slenyto), insomnia with Smith–Magenis syndrome (Slenyto), insomnia associated with behavioural disorders in children and adolescents (Adaflex)).

86.7% of prescriptions were reviewed for efficacy within 3 months while tolerability (side effects) was reviewed in 46.7%.

The need for continuing melatonin treatment was reviewed annually in 80.8% of cases while tolerability was reviewed in 30.8%.

**Conclusion:** The audit revealed high rates of prescription in certain areas of the county, it also showed that documentation of indication and target symptoms was not always available, similarly review of tolerability (side effects) was not always available.

The findings were presented to the CAMHS consultants. The high rates were thought to be related to shift in practice over time, perhaps due to consultants shortage.

Documentation of efficacy was more often done than review of tolerability. One reason for this could be that melatonin was being monitored by the community paediatrics team or the GP.

The need for clear documentation can therefore not be overemphasized.

The audit did not consider those who were able to stop melatonin. This could be useful to support patients.

